# Editorial: The Pupil: Behavior, Anatomy, Physiology and Clinical Biomarkers

**DOI:** 10.3389/fneur.2020.00211

**Published:** 2020-04-09

**Authors:** Andrew J. Zele, Paul D. Gamlin

**Affiliations:** ^1^Visual Science and Medical Retina Laboratories, School of Optometry and Vision Science, Institute of Health and Biomedical Innovation, Queensland University of Technology (QUT), Brisbane, QLD, Australia; ^2^Department of Ophthalmology and Visual Sciences, University of Alabama at Birmingham, Birmingham, AL, United States

**Keywords:** intrinsically photosensitive retinal ganglion cells, autonomic, central nervous system, attention, cognitive modulation, sleep, pupillography, pupillometry

The pupil response is more than a simple light evoked reflex (1). At any moment, pupil diameter reflects the activity of complex neurological pathways to changes in the environmental illumination and autonomic activity through parasympathetic and sympathetic innervations (2). A mobile pupil also modulates retinal illumination and enhances visual performance by affecting the depth of focus and optical aberrations. This special issue brings together 110 co-authors from 17 countries across 24 original research articles and reviews, that together highlight the latest research on the afferent and efferent pupil control pathways in humans and animals and the influence of non-photic control factors on the pupil response, including cognition and attention, sleepiness, and circadian processing. It includes a significant focus on the non-invasive measurement of the pupil as a clinically important neurological marker of autonomic, midbrain, and central brain function. The publications are organized in this eBook according to studies describing the cognitive/sleep-related and light-evoked behavior of the pupil, the anatomy and physiology of pupillary responses, and clinical pupil biomarkers, and begins with the international Standards in Pupillography.

## Standards in Pupillography: Kelbsch et al.

The widespread application of pupillometry in basic and clinical measurements of humans and animals in ophthalmology, neurology, neuroscience, psychology, and chronobiology has necessitated the demand to introduce a set of recommendations and general standards. With this in mind, experts convened at the 32nd International Pupil Colloquium (IPC) in Morges Switzerland to discuss and prepare the first iteration of an international standard for pupillography (Kelbsch et al.). This living standard considers the procedures relating to data collection, processing and a minimum set of variables for reporting in publications. The guidelines cover specific applications, including the afferent pupil light response and conditions for differentiating the pupil light reflex initiated by rhodopsin-driven rod responses, opsin-driven cone responses, and/or melanopsin-driven ipRGC responses, the efferent pupillary pathway, pharmacological effects on the pupil, pupillography in psychology and psychiatry, and methods for evaluating sleepiness-related pupillary oscillations. The standard is applicable to measurements of the pupil in humans and animals and designed to facilitate its correct application and improve the comparability between studies.

## The Pupil: Light-Evoked Responses

In the past 20 years, it has been recognized that the pupillary light reflex is driven predominantly by a unique subset of intrinsically-photosensitive retinal ganglion cells (ipRGCs) that contain melanopsin and project to the pretectum, specifically the olivary pretectal nucleus (3, 4). Further, ipRGCs are strongly influenced by rod and cone inputs in addition to their slower, melanopsin-driven intrinsic responses (5, 6). Thus light-evoked pupillary responses are dependent on both spectral and temporal stimulus characteristics as well as on stimulus intensity (7).

Crippa et al. report original findings in an evaluation of chromatic pupil light responses under dark- and light-adapted conditions in healthy children aged 3–18 years; in their pediatric sample, the amplitude of the melanopsin-mediated pupil response was independent of age; together with previous evidence, melanopsin function is stable between the first and eight decades of life after which dysfunction becomes apparent. This contrasts with the earlier onset of age-related declines in rod and cone photoreceptor density, and highlights the value of measures of melanopsin-mediated pupil function as a clinical biomarker. The authors stratified their sample at 10 years of age and revealed the younger age group had smaller dark-adapted baseline pupil diameters and higher stimulus thresholds for evoking a criterion pupil response compared to the older pediatric group, with the older group being more similar to adults. Crippa et al. infer that the age-related threshold pupil response might reflect decreased retinal input to the olivary pretectal nucleus due to continuing post-natal retinal development in the younger cohort.

Bonmati-Carrion et al. evaluated the effects of extended (5 min) exposure to high irradiance monochromatic and bi-chromatic (polychromatic) stimuli on the pupil light reflex. Their novel evaluation of the pupil response to polychromatic lights was designed to differentially potentiate a change in the conformational state of the melanopsin photopigment; the pupil amplitudes were not however significantly different, indicating that any effect of the putative bistability of the melanopsin photopigment is not manifest in the pupil response under such conditions. On the other hand, the sustained post-illumination pupil response (PIPR) constriction amplitude increased when the monochromatic and polychromatic stimulus lights had higher levels of melanopsin excitation, consistent with literature reports.

Formalized by Estévez and Spekreijse (8) as a method to study the mechanisms of color and luminance processing, silent substitution is now widely applied in human visual neuroscience when pharmacological or transgenic manipulations are not applicable. As such, it is an essential technique for investigating photoreceptor control of the afferent pupillary response in humans, including in two original research articles reported in this special issue. Here, Spitschan and Woelders provide a tutorial on the silent substitution technique for generating metameric stimulus lights that preferentially activate one, or a combination of photoreceptor classes.

Using a multi-primary silent-substitution method to generate rod- and cone-pathway directed stimulus activations, Barrionuevo et al. quantified the summation characteristics of outer retinal photoreceptor inputs to the pupil control pathway. An electroretinogram (ERG) provided a direct measure of outer-retinal signaling and was recorded under the same conditions as the pupillary response. The authors observed that ERG and pupil measurements to photoreceptor-directed stimulus pairs of different temporal frequencies contained a response component at a frequency corresponding to the difference of the stimulus frequencies; this so-called beat response signifies the presence of non-linear rod and cone inputs to the pupil control pathway that originate in the outer retina.

To isolate interactions between melanopsin and the L-, M-, and S-cone-photoreceptor inputs to the afferent pupil light response in trichromatic humans, Zele et al. separated the component photoreceptor inputs using silent substitution and 5-primary photostimulation methods. The authors revealed the melanopsin-mediated pupil response signature as having a long latency and slow constriction velocity that remained sustained during and after stimulus exposure; cone mediated pupil responses had shorter latencies and faster constriction velocities to stimulus onset and rapidly redilated to baseline. Together, the inner and outer retina pupil signals combine additively to set a unified pupil diameter. Cone inputs control the tonic constrictions to variations in stimulus contrast and melanopsin inputs set the light adapted pupil diameter during prolonged light exposures.

The loci of gain-control processes within the pupil pathway that modulate constriction amplitude were identified by Carle et al. using their multifocal pupillographic objective perimetry (mfPOP) technique. The authors examined the pupil constriction to stimuli with different spatio-temporal densities originating from localized hemifields under monocular or binocular viewing. Pupil constriction amplitudes differed only when the signal density differed at the level of Edinger-Westphal nuclei (or later) but not in the retina and pretectal olivary nuclei, and for nasal and temporal hemifield stimulation this trend was present. They infer that pupillary gain controls are present in the Edinger-Westphal nucleus.

## The Pupil: Cognition/Sleep

While many readers will be familiar with the constriction of the pupil that occurs with light, the pupil is also modulated by other factors including cognition, sleep, and arousal (9). For example, many studies have documented that pupil dilation accompanies mental effort or increased attention, while pupils constrict with sleepiness. Further, pupil dilation is seen when the subject experiences heightened vigilance and arousal. In their review article, Ebitz and Moore evaluated the pupil as a peripheral measure of cortical processing. They contend that top-down modulation of pupil diameter, whether it be due to shifts in visual attention or cognition, can cause an active filtering of the incoming light signal that gives rise to functional benefits. Following this, the Research Topic includes two original articles investigating top-down modulation on the pupil.

The effect of autonomic arousal on pupil size during presentation of human faces expressing different emotive content was investigated by Wang et al. In their study, activity of the sympathetic and parasympathetic branches of the autonomic nervous system were recorded concurrently with pupil size, using the galvanic skin response and heart rate. The authors reported a trial-by-trial fluctuation in pupil size prior to the presentation of the face that correlated with their sympathetic and parasympathetic measures. They infer that arousal levels involuntarily regulated by the autonomic nervous system can be indexed by pupil size.

In a study of the role of visual awareness on pupil constriction to scene images under conditions designed to influence top-down processing. Sperandio et al. modulated a person's awareness of images evoking the construct of perceived brightness, such as a picture of the sun. In their paradigm, visual awareness was altered using an interocular flash suppression paradigm. It was found that pupillary constrictions occurred in response to scenes containing images of the sun, but only when participants were visually aware of the image content. The authors suggest that extra-retinal pathways are driving these pupil responses.

The relationship between baseline pupil diameter and sleepiness was investigated by Daguet et al. in a demanding 56 h protocol that included a 36 h period of constant routine in dim light with enforced wakefulness. The baseline pupil diameters decreased linearly with time awake and had a superimposed sinusoidal rhythm wherein diameters were smallest in the morning and largest in the evening. Sleepiness also increased linearly with time awake due to an accumulation of sleep pressure, whereas the circadian drive for sleep followed a sinusoidal process that was phase shifted relative to the circadian variation in pupil diameter. Together these outcomes demonstrate that baseline pupil diameter is applicable as an index of sleepiness only at certain times of the day because of the interactions between the dual regulation of sleepiness by homeostatic and circadian processes.

Using virtual reality displays to generate rapid or gradual shifts in binocular disparity, Balaban et al. characterized the pupil responses and convergent or divergent movements required to resolve diplopia. The resultant eye movements and pupillary responses involved successive epochs of uncorrelated activity, coordinated near response activity and a coordinated opposite response pattern. The authors propose a system in which the disparity-driven ocular and pupillary responses are coordinated by the real-time interactive selection of different modes of a modified disparity controller with separate drives responsive to blur, binocular disparity and global luminance, and an additional three-state gain selection switch.

## The Pupil: Anatomy and Physiology

The final efferent pathways controlling pupil diameter are comprised of both the parasympathetic and the sympathetic components of the autonomic nervous system. Parasympathetic postganglionic neurons project to the sphincter pupillae muscle of the iris to produce pupil constriction, while sympathetic postganglionic neurons project to the dilator pupillae muscle of the iris to produce pupil dilation (2). The development of the afferent pathways controlling the pupil light reflex have not previously been well-studied, and the predominant involvement of the ipRGCs in the reflex had only previously been addressed in rodents following ipRGC elimination (10). Here, Szabadi reviews the functional organization of the sympathetic pathways controlling the pupil with an emphasis on the anatomy and physiology of light-inhibited and light-stimulated pathways, sleep and arousal in nocturnal and diurnal species.

To investigate intrinsically photosensitive Retinal Ganglion Cell (ipRGC) mediation of the pupillary light reflex in Rhesus monkey, Ostrin et al. developed a melanopsin-directed immunotoxin that was delivered intravitreally. With increasing immunotoxin concentration, the pupil constriction to narrowband pulsed lights showed a progressive amplitude decrease and the PIPR was eliminated; at the highest concentration, flicker pupil responses were confined to irregular, transient constrictions. Taken together, the ipRGCs form the primary afferent pathway for mediating pulsed and flicker pupil response in non-human primates. The melanopsin-directed immunotoxin provides a new technique to the study the role of ipRGCs in circadian rhythms, and melanopsin contributions to image-forming visual functions in non-human primates.

The maturation of the pupillary response in two mouse models (C57BL/6 and Sv129S6) was evaluated by Kircher et al. Retinal structure was quantified using immunohistochemistry analysis and the functional PLR measures were combined with electroretinography. Age-related differences in transient and steady-stated pupil diameters were observed during early adulthood (1, 2, and 4 months). Developmental changes in the PLR in C57BL/6 mice were associated with differences in retinal sensitivity related to the rod and cone photoreceptors; in Sv129S6 mice, age-related changes in the PLR may involve variations within the central and/or peripheral pathways controlling the pupil. The authors infer that the circuitry associated with, rods, cones and ipRGCs reach functional maturity in the pupil pathways in adulthood (>2 months of age).

## The Pupil: Clinical Biomarkers

Pupillometry outcomes provide clinical biomarkers of many ophthalmic and systemic diseases (11). Chromatic pupillometry is especially valuable due to its capacity to preferentially separate outer retinal (rod and cone-mediated) and inner retinal (melanopsin) responses in a single, objective, non-invasive pupil measurement (12). Rukmini et al. have reviewed chromatic pupillometry methods currently used for measuring inner and outer retinal photoreceptor function in ophthalmic disease. Further optimizations in these pupillometric technologies will lead to highly sensitive and accurate markers for use in disease detection and for monitoring progression, especially for they provide a direct measure of melanopsin-mediated ipRGC function. La Morgia et al. reviews the clinical studies of melanopsin retinal ganglion cell function in neurological and neuro-ophthalmic conditions, and emphasized its relevance as a biomarker in neurodegenerative disorders in which patients experience sleep and circadian dysfunction. Following this, Chougule et al. consider in their review a specialist application of the pupillary light response as a diagnostic indicator of Alzheimer's disease related effects on sympathetic and parasympathetic nervous system function, and in Parkinson's disease.

Omary et al. consider in their original clinical study the difficulty in diagnosing Horner's syndrome when topical pharmacological test results are inconclusive. A framework is introduced that uses surface electrical stimulation of the median nerve to accentuate the inter-ocular asymmetry of sympathetic innervation to the iris dilator and distinguish healthy from Horner's syndrome patients. In people with an ocular sympathetic deficit, anisocoria during the evoked pupil dilation is enhanced when electrical stimulation is combined with the pupillometry measurement at 2 s after light offset. Compared to a non-electric stimulation pupillometry paradigm, all patients with Horner's syndrome and those with pharmacologically induced Horner's syndrome demonstrate increased anisocoria, whereas in the in healthy participants there was no significant change in anisocoria.

In a retrospective analysis of the results from 660 pharmacological tests using topical administration of cocaine (2–10%) and apraclonidine (0.5–1.0%) in suspected cases of Horner's syndrome, Bremner determined the sensitivity of each drug test for detecting Horner's syndrome. Accounting for the pupil diameter in the dark and light, iris color and age, the sensitivity of apraclonidine for the detection of Horner's syndrome was 93% (criterion for abnormal: mydriasis ≥0.1 mm when measured in the dark), compared to 40% for cocaine (criterion for abnormal: mydriasis ≤ 0.5 mm when measured in the dark).

Dysfunctional ipRGCs can cause aberrant signaling of the ambient illumination to alter photoentrainment and mood in patients with seasonal affective disorder (SAD). Here, Feigl et al. quantified melanopsin function and light exposure in people with non-seasonal major depressive disorder who live in a sub-tropical environment. Compared to age-matched controls, people with major depression had similar melanopsin function and light exposure during a 2-weeks measurement period. The implication is that in seasonal and non-seasonal depressive disorders, the effect of light on mood is likely to be modulated by different pathomechanisms and/or involve different ipRGC subtypes.

To investigate cortical innervation of the pupil pathway via the insular cortex and prefrontal eye field, Peinkhofer et al. measured ipsilateral pupil light responses in a human clinical model with patients having localized ischemic infarcts in these brain areas. In both the patient and control groups, pupil diameter and constriction velocity were positively correlated, and within normal physiological limits. The absence of cortical input to the pupils due to localized damage in the insular cortex or prefrontal eye field therefore does not appear to affect pupil diameter or constriction velocity.

Naber et al. introduced a gaze-contingent flicker pupil perimetry method to objectively record pupil oscillations across the central visual field. In a clinical sample of patients with glaucoma or cerebral visual impairment, the pupil oscillation amplitudes were lower in visual areas having reduced sensitivity on standard automated perimetry. The outcomes provide the initial evidence of the potential diagnostic effectiveness of gaze-contingent flicker pupillometry in quantifying visual defects routinely evaluated in clinical settings using subjective visual perimetry.

As an indicator of autonomic function and trigeminal-vascular system activation in people with migraine, Cortez et al. measured the afferent-efferent pupillary light circuit using the edge-light pupil cycle time. This pupil cycle time was sufficiently sensitive so as to distinguish each migraine severity group from the non-headache controls. These findings reveal a potential opportunity for application of the edge-light test recorded under slit lamp examination as a simple test for detecting the earliest stages of peripheral trigeminal sensitization.

This special issue displays the diversity of the basic and clinical research currently undertaken to understand the behavior, anatomy and physiology of the pupil control pathway. The introduction of an international standard, and the development of new pupillometry methods will facilitate its widespread translation to clinical practices for the detection and monitoring of neurological disorders, for use as a biomarker in clinical trials for objective assessment of autonomic nervous system activity and as a direct measure of inner retinal (melanopsin) and outer retinal (rhodopsin and cone-opsin) mediated function. Studies show that melanopsin expressing ipRGCs form the primary afferent pathway for the pupil light response in mice (13) and primate (14, 15). The ipRGCs have reduced redundancy compared to conventional ganglion cells (5), are more resistant to age related decline than cells within the conical retinogeniculate pathways (16, 17), transmit information for image-forming vision (18) and for light dependent non-mage forming circadian and mood (19), and can be quantified directly in a single unitary measure, through pupillometry (12, 20). As a gateway to the central and peripheral nervous systems, the pupil has much to reveal to all those who study it.

## Author Contributions

All authors listed have made a substantial, direct and intellectual contribution to the work, and approved it for publication.

### Conflict of Interest

The authors declare that the research was conducted in the absence of any commercial or financial relationships that could be construed as a potential conflict of interest.
